# Pleiotropic ZmICE1 Is an Important Transcriptional Regulator of Maize Endosperm Starch Biosynthesis

**DOI:** 10.3389/fpls.2022.895763

**Published:** 2022-07-22

**Authors:** Hanmei Liu, Yongbin Wang, Lijun Liu, Bin Wei, Xieqin Wang, Qianlin Xiao, Yangping Li, Babatope Samuel Ajayo, Yubi Huang

**Affiliations:** ^1^College of Life Science, Sichuan Agricultural University, Ya'an, China; ^2^College of Agronomy, Sichuan Agricultural University, Chengdu, China; ^3^College of Agronomy and Biotechnology, Southwest University, Chongqing, China; ^4^State Key Laboratory of Crop Gene Exploration and Utilization in Southwest China, Sichuan Agricultural University, Chengdu, China

**Keywords:** maize, starch biosynthesis, ZmICE1, transcriptional regulation, co-expression

## Abstract

Starch, the major component of cereal grains, affects crop yield and quality and is widely used in food and industrial applications. The biosynthesis of maize starch is a complex process involving a series of functional enzymes. However, the sophisticated regulatory mechanisms of starch biosynthetic genes have not been fully elaborated. The basic/helix-loop-helix (bHLH) transcription factors are widely distributed in eukaryotes and participate in many physiological processes. In this study, 202 bHLH encoding genes were identified in the maize genome by Blast method. *ZmICE1* gene, which belongs to the ICE subfamily of the bHLH family, was obtained and expressed mainly in maize filling endosperm and co-expressed with 14 starch biosynthesis genes. Based on the comparative analyses across different plant species, we revealed that the gene structures and protein domains of the ICE subfamily were conserved between monocots and dicots, suggesting their functional conservation feature. Yeast activation and subcellular localization assays suggested that ZmICE1 had transcriptional activation activity and localized in the nucleus. Yeast one-hybrid assays confirmed that ZmICE1 could directly bind to the promoters of *ZmSSIIa* and *ZmGBSSI*. Transient gene expression analysis in maize endosperm revealed that ZmICE1 positively regulated the expression of *ZmSSIIa*, but inhibited the expression of *ZmGBSSI*. Our results indicated that ZmICE1 could function as a regulator of maize starch biosynthesis.

## Introduction

Starch is the main component of cereal endosperm and is also a key factor that influences crop yield and quality. Starch accounts for about 70% of maize grain weight and is widely used for human food, animal feed, fuel and industrial raw materials. So, it is crucial to study the mechanism of maize starch biosynthesis. Starch is synthesized in the plastids of higher plants through the concerted activities of four key enzymes, including ADP-glucose pyrophosphorylase (AGPase), starch synthase (SS), starch branching enzyme (SBE) and starch debranching enzyme (DBE) (Myers et al., 2000; James et al., 2003; Tetlow et al., 2004; Hennen-Bierwagen et al., 2009). The formation of glycosyl donors, ADP-glucose (ADPG), is catalyzed by AGPase; a rate-limiting step in the starch biosynthesis pathway (James et al., 2003). SS catalyzes the glucan chain extension by adding a glucose unit (donated from ADPG) to the non-reducing end of the acceptor chains (Ball and Morell, 2003; Keeling and Myers, 2010). Starch synthases are classified into two groups, granule-bound starch synthase (GBSS) and soluble starch synthesis (SSS), which are mainly responsible for the synthesis of amylose and amylopectin, respectively (Delrue et al., 1992). SBE catalyzes the formation of the branch linkage by cleaving the existing α-1,4-glucan chains and reattaches them to the glucosyl residue of another (or the same) glucan chain through α-1,6-linkage (Zeeman et al., 2010). DBE, classified into isoamylase and pullulanase, are strongly implicated in amylopectin synthesis for the hydrolysis of disordered branched linkages and also involved in the formation of starch granule and the degradation of starch (Myers et al., 2000; Burton et al., 2002; Nakamura, 2002; Zeeman et al., 2007).

Starch synthesis is not only coordinated by multiple enzymes, but also depends on the transcriptional regulations. Transcription factors and *cis*-acting elements have become important components of transcriptional regulation in starch biosynthesis (Sun et al., 2003; Fu and Xue, 2010; Hu et al., 2011; Wang et al., 2013). HvSUSIBA2, a WRKY family transcription factor in barley, can directly bind to the sugar-responsive elements in the *ISO1* promoter and served as an activator in the transcriptional regulation (Sun et al., 2003). OsbZIP58 can directly bind to the promoters of *OsAGPL3, Wx, OsSSIIa, SBE1, OsBEIIb* and *ISA2* and regulates their expressions (Wang et al., 2013). ZmABI4 mediates the expression of *ZmSSI* gene in maize through ABA-induction by binding to the CACCG motif (Hu et al., 2012). ZmbZIP91 regulates starch synthesis by binding to the ACTCAT element in the promoters of starch biosynthetic genes in maize (Chen et al., 2016).

The basic/helix-loop-helix (bHLH) proteins, a superfamily of transcription factors in eukaryotes, are defined by the bHLH signature domain (Riechmann et al., 2000). The bHLH domain contains two functionally distinct amphipathic-helices separated by a loop region of variable sequence and length, and is located at the C-terminal of the protein (Toledo-Ortiz et al., 2003; Li et al., 2006). bHLH proteins are essential regulators and are widely involved in plant physiological pathways (Yadav et al., 2005; Zhou et al., 2009). Meanwhile, bHLH proteins also play vital roles in the regulation of cell size, vegetative biomass, reproductive yield, flavonoid biosynthesis and endosperm development in plant (Hichri et al., 2010; Lim et al., 2018; MacGregor et al., 2019). Opaque 11, an endosperm-specific bHLH transcription factor in maize, was found to directly regulate key transcription factors including ZmDof3, O2, and PBF (Feng et al., 2018). ZmDof3, O2, and PBF have been reported to participate in the regulation of maize starch synthesis (Zhang et al., 2016; Qi et al., 2017), suggesting that Opaque 11 also participate in the transcriptional regulation of maize starch biosynthesis.

Gene co-expression analysis is widely used to screen candidate genes involved in specific physiological and metabolic processes (Persson et al., 2005; Aoki et al., 2007). Co-expression of genes has been reported in starch biosynthesis pathway in maize (Giroux et al., 1994), *Arabidopsis* (Li et al., 2007; Tsai et al., 2009), rice (Fu and Xue, 2010) and potato (Ferreira et al., 2010). Some transcription factors involved in the regulation of starch biosynthesis have been identified through co-expression analysis. For example, OsRSR1 co-expresses with some starch biosynthesis-related genes and negatively regulates these genes in rice endosperm (Fu and Xue, 2010). A maize NAC transcription factor was identified and associated with starch synthesis by co-expression analysis (Zhang et al., 2014).

In this study, we found two transcription factors, ZmbHLH167 and ZmbHLH175, highly expressed in maize endosperm and co-expressed with genes related to starch synthesis, which are Opaque 11 and ZmICE1, respectively. Opaque 11 is a central hub of the regulatory network of maize endosperm, linking development, nutrition metabolism and stress responses (Feng et al., 2018). ICE1 can interact with O11/ZmZHOUPI, and be inferred to regulate abiotic stress in endosperm (Grimault et al., 2015; Feng et al., 2018). ICE1 was also identified as a target for an eQTL, which was a master epi-transcriptomic regulator of endosperm development (Pang et al., 2019). Recent reports showed that ICE1 had pleiotropic effects in plants including regulation of freezing tolerance, determining the depth of primary seed dormancy and increasing yield (Chinnusamy et al., 2003; Lu et al., 2017; MacGregor et al., 2019; Verma et al., 2020). However, no information linking ICE1 and starch synthesis has been reported. We found that *ZmICE1* had more transcripts in maize endosperm than other tissues, and was co-expressed with genes related to starch synthesis. Meanwhile, ZmICE1 could bind directly to the promoters of starch synthesis genes in yeast, and regulated the promoter activities of *ZmSSIIa* and *ZmGBSSI* in maize endosperm. Thus, ZmICE1 may play a key role in the regulation of starch biosynthesis in maize endosperm.

## Materials and Methods

### The Identification, Phylogenetics and Expression Analysis of Maize bHLH Family Genes

The basic/helix-loop-helix (bHLH) proteins are a superfamily of transcription factors identified in different species (Toledo-Ortiz et al., 2003; Li et al., 2006). To determine all bHLH-related proteins and encoding genes in maize, BLASTp was performed to search against the maize genome dataset (ZmB73_5b_FGS_translations.fasta.gz) with an E <1 ×10^−5^, using rice and *Arabidopsis* bHLH proteins as query sequences. Proteins containing bHLH domain were defined as bHLH superfamily and further verified using SMART (http://smart.embl-heidelberg.de/). The expression data of the bHLH superfamily was mainly based on the RNA-sequence data downloaded from the MaizeGDB (https://www.maizegdb.org/), which included more than 60 distinct tissues representing different development stages of the 11 major organs (Stelpflug et al., 2016). Tbtools (Chen et al., 2020) was used to build expression heat maps.

### Identification of Candidate bHLH Genes Associated With Starch Biosynthesis

Co-expression analysis was performed based on the method of Fu and Xue (Fu and Xue, 2010). Twenty-seven highly expressed starch biosynthetic related genes in maize endosperm were considered as guide genes (Nelson and Pan, 1995; James et al., 2003). The Pearson Correlation Coefficients (PCC) between the transcription factors of the bHLH family and guide genes were calculated based on the RNA-sequence data. If the absolute value of a PCC is >0.60, the probe will be considered to be associated with the guide gene. The genes associated with more than six guide genes were classified as candidate factors involved in maize starch biosynthesis.

### Plant Materials and Growth Condition

Maize inbred line Mo17, provided by the Maize Research Institute of Sichuan Agricultural University (SAU), was grown on the Research Farm of SAU in Wenjiang according to the agronomic guidelines. The roots, stems and leaves were obtained after the maize was in the initial jointing stage of growth. Anthers were collected during the dispersal period, and silks were obtained before emerging from the husks. Kernels of 15 days after pollination (DAP) were collected for semi-quantitative RT-PCR analysis. For qPCR analysis, whole kernel, embryo and endosperm were obtained at different development stages after pollination. All tissue samples were immediately frozen in liquid nitrogen and stored in a refrigerator at −70°C for use in further experiments.

### Candidate Gene Cloning and Sequence Analysis

Total RNA was isolated using TRIzol reagent (Invitrogen) for specific tissues of the Mo17 according to the manufacturer's instructions. First-strand cDNA was synthesized from 1.5 μg of total RNA using the PrimeScript^TM^ RT reagent Kit with gDNA Eraser (TaKaRa, Japan). According to RNA-sequence information, the full-length cDNA of ZmbHLH175 (*ZmICE1*) was cloned from maize seed at 15 DAP. The sense and anti-sense primers were bHLH175F: 5'- TCAGGATCCATGGACGACTCGG-3' and bHLH175R: 5'-GAGCTCCTACATTGCGTTGTGGAG-3'. The PCR was performed under the following conditions: 20 μL of reaction mixture containing 10 μL 2^*^KOD Buffer, 3 μL dNTPs, 0.3 μL KOD Neo, 1 μL cDNA template, 4.7 μL H_2_O, 0.5 μL bHLH175F and 0.5 μL bHLH175R, was pre-denatured at 94°C for 4 min, followed by 35 cycles of 30 s at 94°C, 30 s at 60°C and 90 s at 72°C. At least three independent PCR replicates were performed. The PCR products were fractionated on 1.5% agarose gel containing GoldView™ and photographed under UV light. Genomic DNA of inbred line Mo17 was used as the template to clone the promoters of starch synthesis genes. All gene fragments were cloned into pMD19-T vectors (Takara, Dalian, China) by using KOD enzymes (Toyobo, Osaka, Japan) for sequencing.

Using ZmICE1 as query sequence, the full-length protein sequences of ICE subfamily in several plant species, including *Arabidopsis*, rice, maize, sorghum, and foxtail millet, were identified by Blastp. Multiple sequence alignment and phylogenetic tree construction were implemented in MEGA7.0 by the neighbor-joining method (Kumar et al., 2016). Bootstrapping test of tree was performed using 1,000 sampling repetitions. The syntenic relationships of the genes of several plants were analyzed by Tbtool. Exon/intron structures were analyzed using the GENE Structure Display Serve 2.0 (http://gsds.cbi.pku.edu.cn/). The conserved domains of the putative ICE proteins were identified by searching against the NCBI's conserved domain database (https://www.ncbi.nlm.nih.gov/Structure/cdd/wrpsb.cgi). The conserved motifs with default parameters were analyzed using MEME (http://meme-suite.org/tools/meme), and the maximum number of motifs was set as 10.

### Expression Analysis of ZmICE1

1.5 μg RNA from different tissues was used as template for the synthesis of the first-strand cDNA, which was used for semi-quantitative RT-PCR and real-time PCR. The sense and anti-sense primers, bHLH175QF: 5'-GCTTCAACCCATCAACACC-3' and bHLH175QR: 5'-CTTCCCTCATCCTGACTTCG-3', were used for semi-quantitative RT-PCR and real-time PCR analysis. The β*-actin* was amplified as the internal control, with primers ACTINF: 5'- CTGGAATGGTCAAGGCTGGT-3' and ACTINR: 5'-CTGGAATGGTCAAGGCTGGT-3'. The PCR was performed using 20 μL of reaction mixture containing 2 × Premix Taq, 8 μL H_2_O, 1 μL template, 0.5 μL bHLH175QF and 0.5 μL bHLH175QR. The reaction mixture was pre-denatured at 94°C for 4 min, followed by 30 cycles of 30 s at 94°C, 30 s at 59°C and 30 s at 72°C. All PCRs were repeated for use for at least three independent samples. The PCR products were fractionated on 1.5% agarose gel containing GoldView™ and photographed under UV light.

The quantitative real-time PCR analysis was performed using the iCycler instrument, model 5.0 (Bio-Rad, Hong Kong), in a total reaction volume 10 μL of SYBR Green PCR Master Mix (TaKaRa, Japan). The Ct value was determined using the instrument's software. The relative quantification of gene expression was monitored after normalization to β*-actin* expression as the internal control. Relative transcription levels were calculated using the -ΔΔCt method based on the internal reference expression of maize β*-actin*.

### Subcellular Localization and Transcriptional Activation Analyses of ZmICE1

The pGBKT7 containing the GAL4 binding domain was used to analyze the transcriptional activity of ZmbHLH175 in yeast system. The ZmbHLH175 was cloned into the pGBKT7 vector with the Sense primer (5'-CATATGGACGACTCGGCGGAG-3') and Anti-sense primer (5'-GGATCCCTACATTGCGTTGTGGAG-3'). The restriction enzyme sites were *Nde*I and *Bam*HI. The completed carrier pGBKT7-ZmbHLH175 was transformed into the yeast strain AH109 to investigate the transactivation of the transcription factor. The transformants were screened on the SD/-Trp plates, grown for 3 days, and mono-clones were picked for propagation in a 2 ml micro-tube. The colonies were further screened on SD/-Trp-His-Ura plates with X-α-gal and cultured at 28°C for 3 days to analyze transcription activation activity.

The pCAMBIA2300-35S-eGFP was used for subcellular localization analysis. ZmbHLH175 with *Kpn*I and *Bam*HI sites was amplified without the termination codon by PCR and cloned into pCAMBIA2300-35S-eGFP. The sense and anti-sense primers were 5'-GGTACCATGGACGACTCGG-3' and 5'-GGATCCCATTGCGTTGTGG-3', respectively. The constructed pCAMBIA2300-35S-ZmbHLH175-eGFP was bombarded into onion epidermal cells through the helium biolistic gun transformation system (Bio-Rad, USA) according to previous method (Hu et al., 2012), and incubated in darkness for 24 h at 28°C. The sub-cellular localization of eGFP fusion proteins was visualized with a florescence microscope ECLIPSE 80i under blue excitation light with 488 nm wave length.

### Particle Bombardment and Transient Expression Assay

The modified pBI221 plasmid, pUbi:GUS, was constructed based on previous method (Hu et al., 2012) and was used for transient expression assay in particle bombardment experiments. pUbi:GUS was used as the internal control. Pro:Adh:LUC (Pro represents the different promoters of genes related to starch synthesis) and Ubi:*ZmICE1* were constructed as the reporter and effector plasmids, respectively. Pro:Adh:LUC contained the Adh1 intron 1 (Adh) between Pro and Luc to enhance promoter activity (Mascarenhas et al., 1990). The promoter fragments were sub-cloned into the pBI221 with the restriction sites of *Bam*HI and *Pst*I, and all the Pro:Adh:LUC vectors with different promoters were provided by the lab. Maize endosperms were isolated from kernels at 10 DAP and cultured on Murashige and Skoog (MS) medium, and were subsequently transfected by particle bombardment biolistic system. The bombarded tissues were incubated for 24 h and the GUS and LUC activities were measured.

### Interaction Between ZmICE1 and the Promoters of Genes Related to Starch Biosynthesis by Yeast One-Hybrid Analysis

Yeast one-hybrid assays were essentially performed to test whether ZmICE1 can bind to the promoters of genes related to starch synthesis. The full length of the promoters and their truncated derivatives were separately cloned into the pHis2 vector with *Eco*RI and *Mlu*I restriction sites named pHis2-promoter (fragment). The CDS sequence of *ZmICE1* was cloned into pGADT7-Rec2 with *Nde*I and *Bam*HI restriction sites named pGADT7-Rec2-*ZmICE1*. The pHis-53 and pGADT7-Rec2-*ZmICE1* constructs were used as a negative control, and pGADT7-Rec2-*ZmICE1* and pHis2-promoter (fragment) constructs as the experimental group. Yeast strain Y187 was used as the host and transformed with the control and experimental constructs. The interaction between promoter (fragment) and ZmICE1 was detected according to the growth status of yeast cells cultured on SD/-His/-Leu/-Trp selective medium with 3-amino-1,2,4-triazole (3-AT) at the final concentration from 50 to 150 mM growth for 3 day.

## Results

### Identification and Expression Pattern Analysis of Maize bHLH

**Figure 1:**
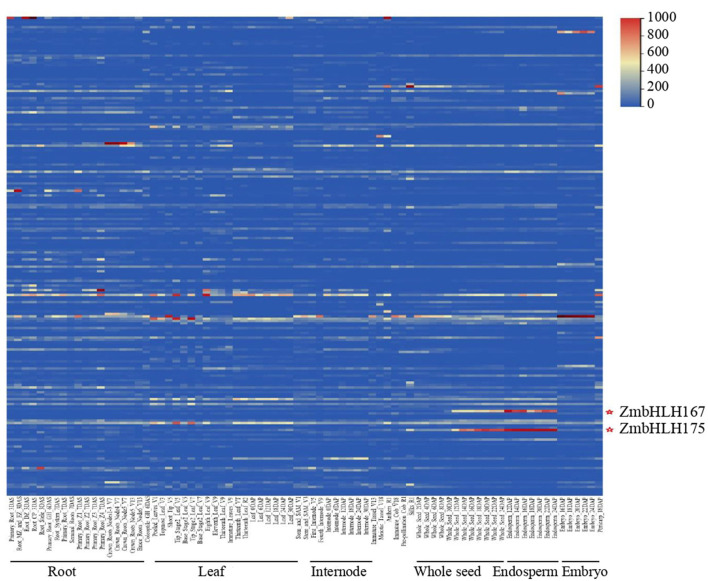
The expression profiles of the *ZmbHLH* superfamily. The expression patterns of 202 *ZmbHLH* genes are showed. Each line represents a ZmbHLH gene, and the expression of ZmbHLH1- ZmbHLH202 is showed from top to bottom successively.

Sequences of bHLH proteins in *Arabidopsis* and rice (Toledo-Ortiz et al., 2003; Li et al., 2006) were used to search the maize genome annotation database by BLASTp. In all, 202 genes encoding the bHLH proteins were identified and named from ZmbHLH1 to ZmbHLH202, including all the 175 annotated ZmbHLHs (from ZmbHLH1 to ZmbHLH175) in the MaizeGDB database (Supplementary Table S1). To investigate the expression pattern of the ZmbHLH superfamily, we analyzed the expression of all ZmbHLH genes based on the RNA-sequence data. The results showed that the genes *ZmbHLH167* (GRMZM2G147685; *ZmO11*) and *ZmbHLH175* (GRMZM2G173534; *ZmICE1*) were significantly expressed in maize seed, especially in seed endosperm (Figure 1). It was suggested that *ZmO11* and *ZmICE1* might exert certain functions in endosperm. Co-expression analyses showed that both *ZmO11* and *ZmICE1* showed a positive co-expression relationship with 14 starch biosynthetic genes, including *ZmGBSSI, ZmSSIIa, ZmBT1* and *ZmBt2* (Supplementary Figures S1A,B, Supplementary Table S2). The result suggests that *ZmO11* and *ZmICE1* may be candidate genes involved in the regulation of starch biosynthesis in maize endosperm. The role of *ZmO11* in developmental seeds as a hub regulator had been recently reported (Feng et al., 2018). However, to date, *ZmICE1* and its orthologous gene in *Arabidopsis* (*AtICE1*) were reported to be involved in abiotic stress processes (Chinnusamy et al., 2003). The role of *ZmICE1* gene in seed development and starch biosynthesis is uncertain yet.

### ICE Subfamily Is Well Conserved in Plants

**Figure 2:**
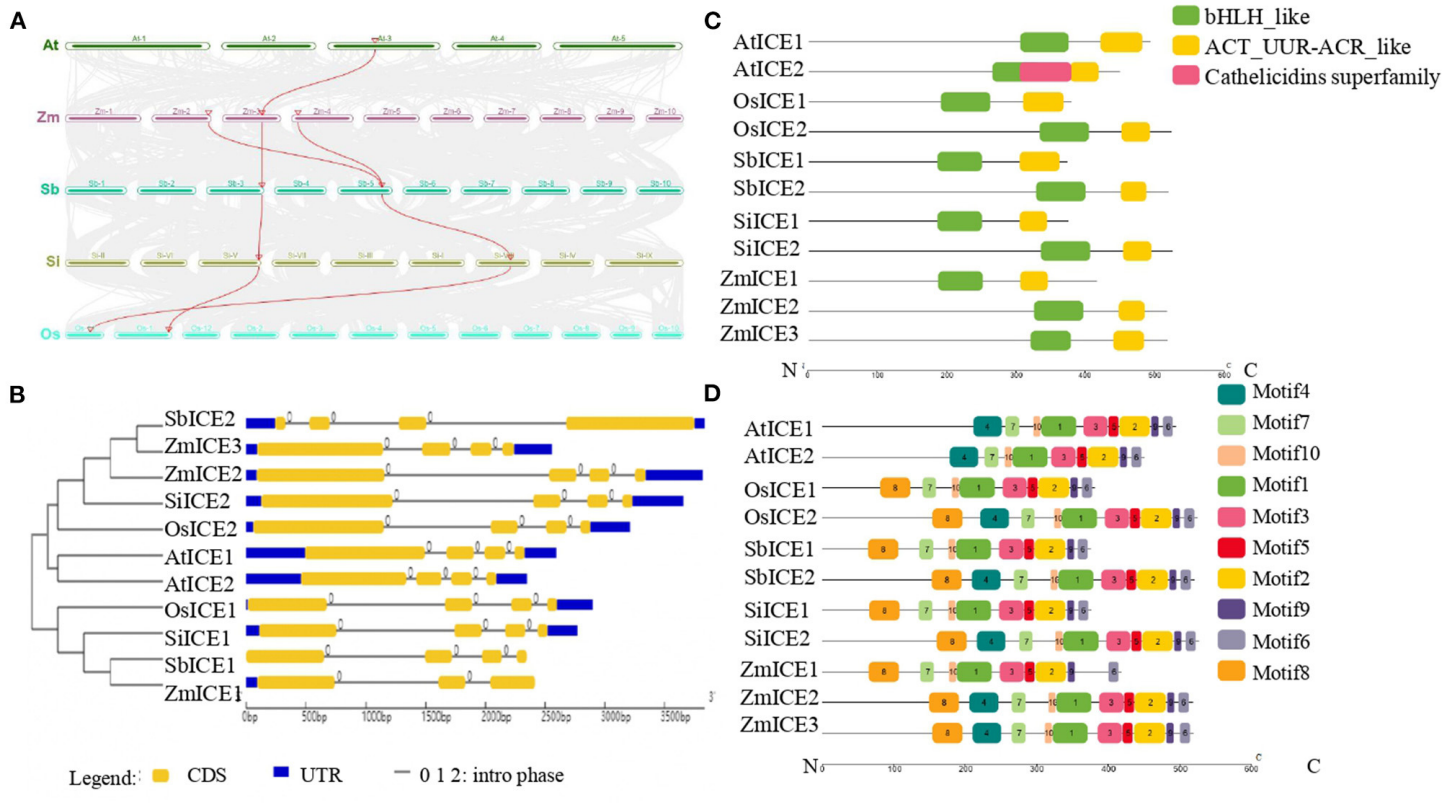
Sequences analysis of the ICE subfamily in plants. **(A)** Syntenic relationships of *ICE* genes in several plants. The chromosome blocks with syntenic relationships are connected by gray lines, and red lines represent *ICE* genes. **(B)** Phylogenetic and gene structure analysis of ICE subfamily. CDS sequences are represented with yellow round-corner rectangles and introns with gray lines, UTRs are shown with blue rectangles. **(C,D)** Domains and motifs analysis of ICE proteins, respectively. Different colored boxes represent the different types of domains and motifs. The abbreviations of plant names were labeled with the prefix of the initials of the genus and species.

Based on the protein sequence of ZmICE1, the ICE subfamily members were identified in 5 plant species, including *Arabidopsis thaliana, Zea mays, Sorghum bicolor, Setaria italica* and *Oryza sativa* (Supplementary Table S3). There are 3 members in maize, and 2 members in each of the other plant species. To reveal the origin of each member, syntenic and phylogenetic relationships were investigated. The member of the *ICE1* gene is located in the syntenic chromosome regions of both monocots and dicots (Figure 2A), instead of *AtICE2*, because *AtICE2* originated from a recent duplication within Brassicaceae (Kurbidaeva et al., 2014). For cereals, the origin of duplication of *ICE1* and *ICE2* was earlier than the species differentiation of Poaceae, so the two members exist in all cereals and formed two main branches in the phylogenetic tree (Figure 2B). In maize, the synteny analysis revealed the possible origin of *ZmICE2* and *ZmICE3* by maize species-specific whole-genome duplication (mWGD) (Wang et al., 2015). We obtained 20 homologous gene pairs that are present in both genomic fragments encompassing *ZmICE2* on chr2 and *ZmICE3* on chr4 (Supplementary Figure S2, Supplementary Table S4). Moreover, the order of the genes and the orientation of the chromosome fragment of the 20 homologous gene pairs are conserved. The gene names and Ks of each gene pair are listed in Supplementary Table S4. The Ks of most gene pairs were similar to that of nucleotide substitution of mWGD. These data indicated that mWGD led to the origin of *ZmICE2* and *ZmICE3*.

The analysis of gene structure revealed that the ICE subfamily genes had three to four exons, and all intron phases were “0.” As expected, most members of the ICE subfamily had similar intron-exon compositions, including the number and length of exons. All ICEs contain two conserved domains, the basic helix-loop-helix (bHLH) domain shown in green, and ACT domains shown in yellow (Figure 2C). The basic region may bind DNA to a consensus hexanucleotide sequence. ACT domains are commonly and specifically involved in the binding of amino acids or other small ligands. MEME analysis of the ICE subfamily proteins identified 10 putative conserved motifs. The number and distribution of motifs in each protein are similar, except that two AtICEs lack motif8 (Figure 2D).

### Cloning and Expression Analysis of *ZmICE1*

**Figure 3:**
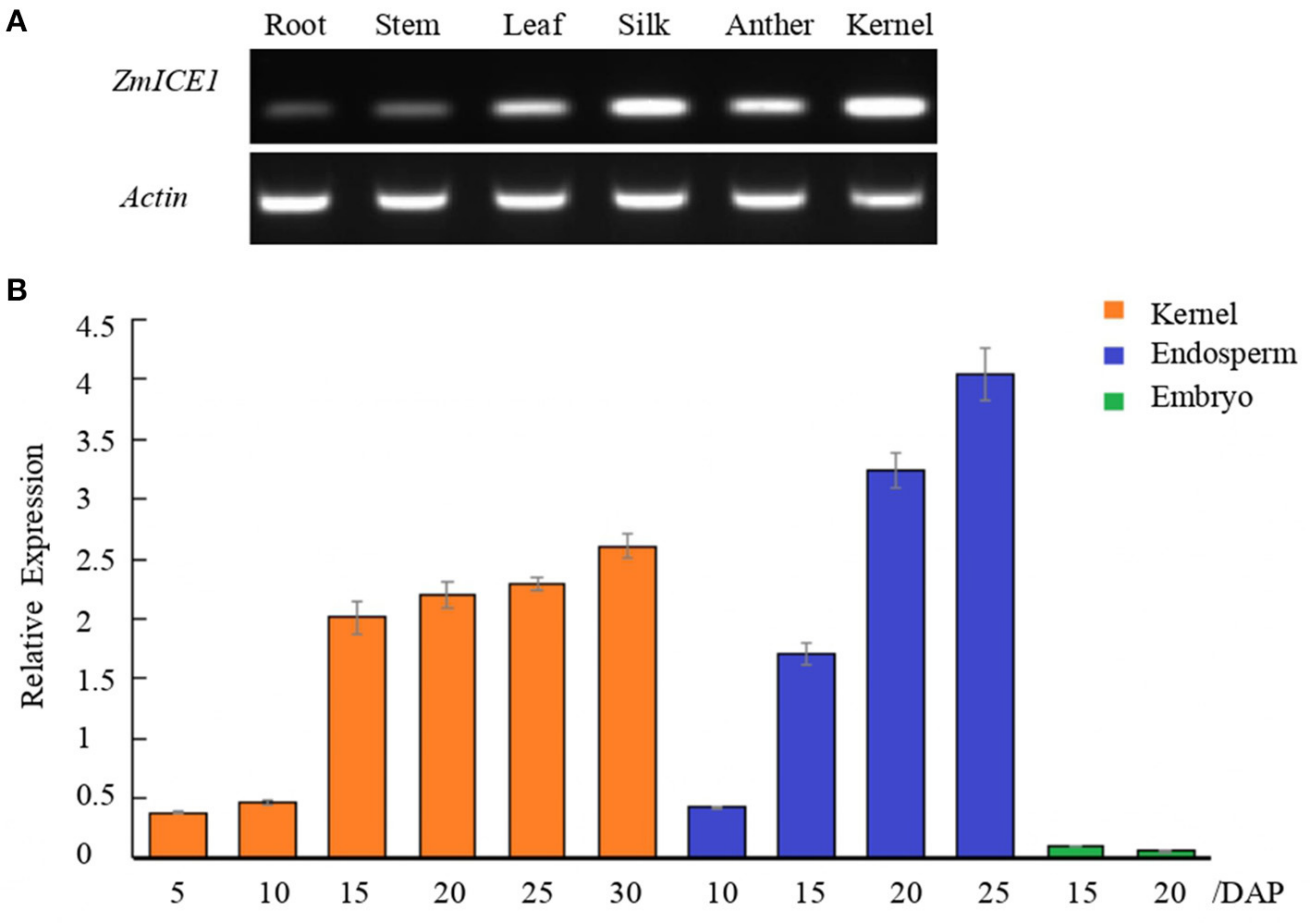
Analysis of *ZmICE1* expression patterns. **(A)** Expression of *ZmICE1* in different tissues by semi-quantitative RT-PCR. **(B)** Expression analysis of *ZmICE1* in maize seeds at different development stages by qPCR. The numbers on the X-axis represent days after pollination (DAP). Y-axis shows the relative expression of *ZmICE1*, which are normalized according to *actin* gene as the internal control.

Based on the reference genome sequence (Jiao et al., 2017), the full-length CDS of *ZmICE1* (1350bp) was amplified by RT-PCR from the maize Mo17 inbred line. The nucleotide sequence of *ZmICE1* was predicted to encode 449 amino acids, which is slightly longer than that of the genome annotation. Semi-quantitative RT-PCR was performed to analyze the expression of *ZmICE1* in different tissues (Figure 3A). The results showed that *ZmICE1* was expressed in all the tested tissues (root, stem, leaf, anther, silk and kernel), but showed a higher expression in seed. Then, the gene expression pattern of *ZmICE1* in maize kernel, endosperm and embryo at different developmental stages was further investigated by quantitative PCR analysis. During the kernel filling period, *ZmICE1* exhibited a high expression level between 15 and 30 days after pollination (DAP). The transcript accumulation was observed mainly in endosperm (not in embryo), with a peak at 25 DAP (Figure 3B). Thus, it is suggested that *ZmICE1* may play an important role in seed endosperm development and also in starch biosynthesis.

### Transcription Factor Characterization of ZmICE1

**Figure 4:**
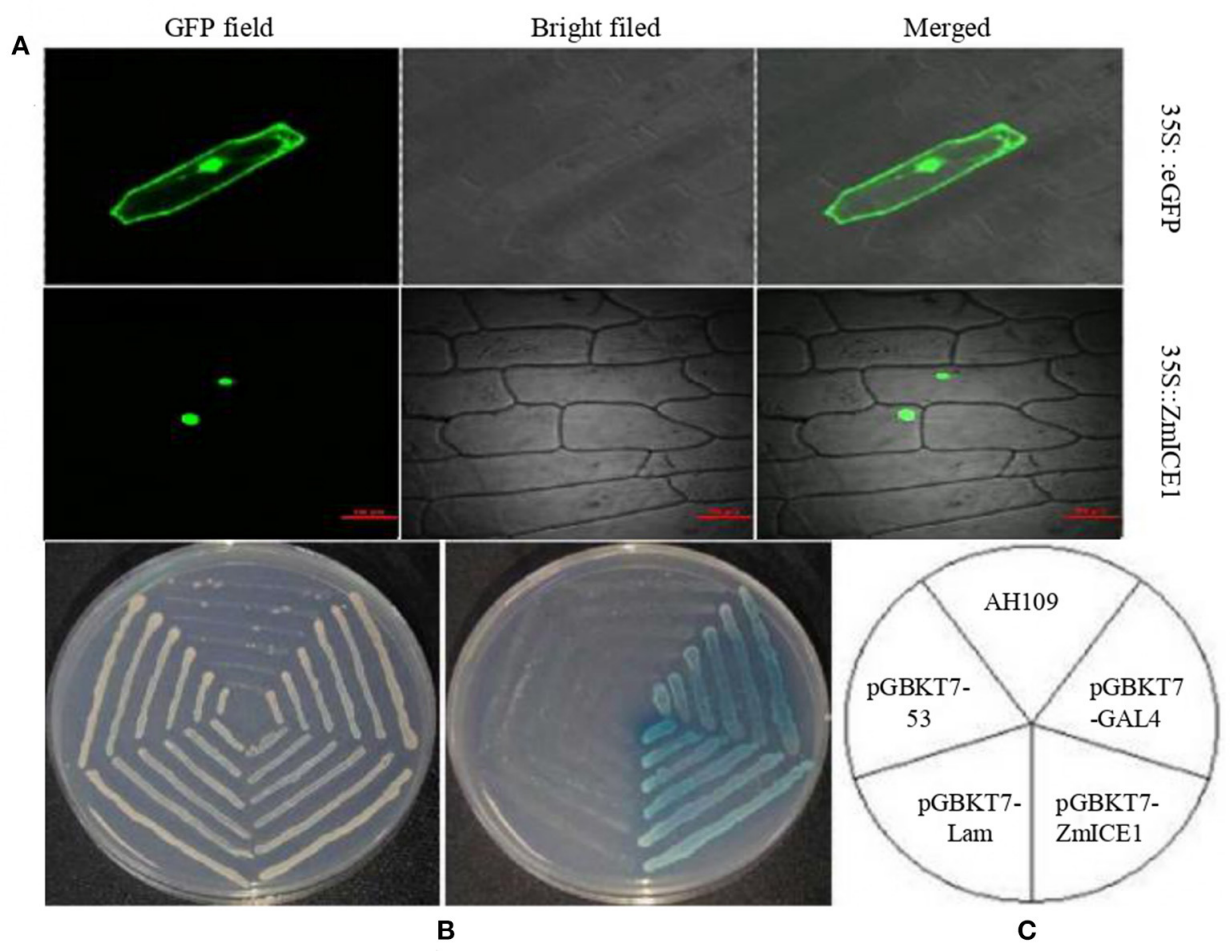
Subcellular localization and transcription activation capacity analysis of ZmICE1. **(A)** Subcellular location of ZmICE1. A GFP fusion protein driven by the 35S promoter was used for analysis by fluorescence microscopy. **(B,C)** Transcription activation analysis of ZmICE1 in yeast AH109. **(B)** Yeast screened on SD/–Trp medium (left) and SD/-Trp-His–Ura medium with X-α-gal (right) in accordance with the structure outlined in **(C)**. **(C)** Schematic diagram of the transcription activity test. AH109 yeast strains was transformed with different constructs. pGBKT7-GAL4 is the positive control; pGBKT7-ZmICE1 represents the experiment group; pGBKT7-Lam and pGBKT7-53 are negative controls; AH109 represents the yeast cells without any vector.

A typical transcription factor contains specific functional domains, such as nuclear localization signal, activation domain, DNA-binding domain and oligomerization sites. The subcellular location of ZmICE1 protein was investigated in onion epidermal cells (Figure 4A). The 35S:eGFP construct was used as a control. As shown in Figure 4A, the *ZmICE1*:eGFP fusion protein was localized in the nucleus, and the 35S:eGFP control was located both in the cytoplasm and in the nucleus, indicating that ZmICE1 was a typical transcription factor and functioned in the nucleus.

The GAL4 system was used to analyze the transactivation activity of ZmICE1. The pGBKT7-Lam and pGBKT7-53 vectors were used as negative controls, and pGBKT7-GAL4 as the positive control. The constructs were transformed into yeast strain AH109 and cultured on the SD medium for detection. As shown in Figures 4B,C, the yeast cells without vector could not grow on the SD/–Trp and SD/–Trp–His–Ura; Negative control transformants could grow on SD/–Trp medium, but were not able to grow on the SD/–Trp–His–Ura and turn blue. The transformants of the positive control and ZmICE1 constructs were able to grow to turn blue on the SD/–Trp–His–Ura. These results suggest that ZmICE1 has transcription activation activity. So it is inferred that ZmICE1 has the potential to promote or inhibit the expression of different target genes, just as ZmbZIP91, which acts as a transcriptional activator of starch biosynthesis (Chen et al., 2016).

### The Interaction Analysis of ZmICE1 and Starch Biosynthesis Gene Promoters by Yeast One-Hybrid

As mentioned above, *ZmICE1* gene shared a positive co-expression relationship with 14 genes related to starch biosynthesis. To explore the potential regulatory role of ZmICE1 in starch biosynthesis, we analyzed the *cis*-elements in the promoter regions (upstream 2000bp of translation start site) of the 14 starch biosynthesis genes by a Python script. The result revealed that most of the promoters of the starch synthesis-related genes contained the consensus sequence “CANNTG” (Supplementary Table S5), which was the potential recognizable binding sites of ICE subfamily (Meshi and Iwabuchi, 1995).

**Figure 5:**
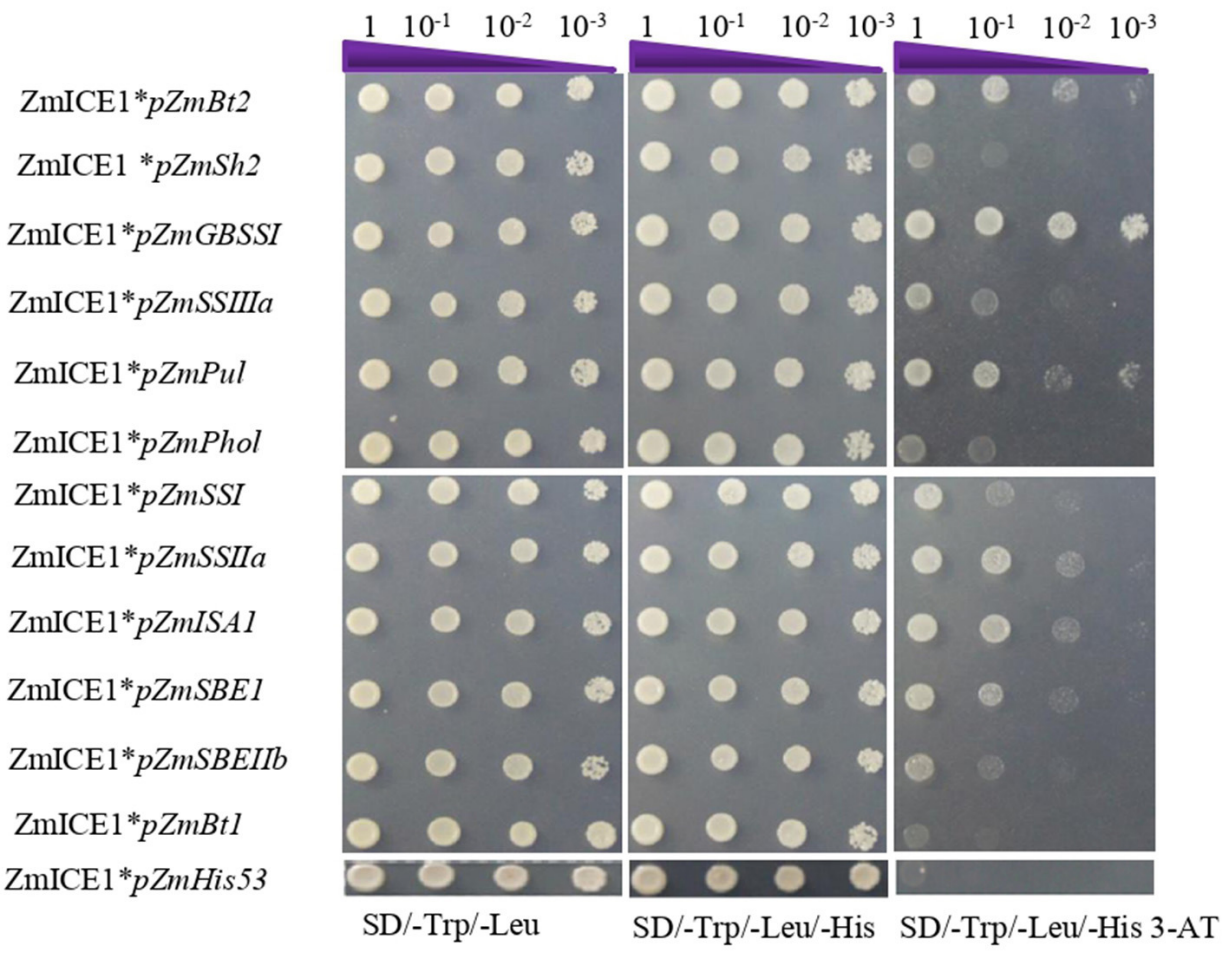
Analysis of ZmICE1 binding to promoters of genes involved in maize starch biosynthesis using yeast one-hybrid assay. pHis-53 was used as the negative control.

Yeast one hybrid assay was then used to analyze the binding of ZmICE1 to promoters of starch synthesis genes. Promoters (upstream 1500–2000bp of the translation start site) of 12 key genes of starch biosynthesis (for example, *ZmBt2* and *ZmSh2*) were cloned and used to evaluate their interaction with ZmICE1 in this study; except for *ZmAGPL3* and *ZmSSV*, whose promoters were not constructed successfully. The constructs, pGADT7-ZmICE1-Rec2 and pHIS2 harboring the 12 promoters, were co-transformed into yeast strain Y187 and screened on the selective media. The results showed that ZmICE1 could directly bind to the promoters of *ZmBt2, ZmGBSSI, ZmPul, ZmSSIIa, ZmISA1*, and *ZmSBEI* (Figure 5).

### ZmICE1 Regulates the Expression of Starch Biosynthesis Genes

**Figure 6:**
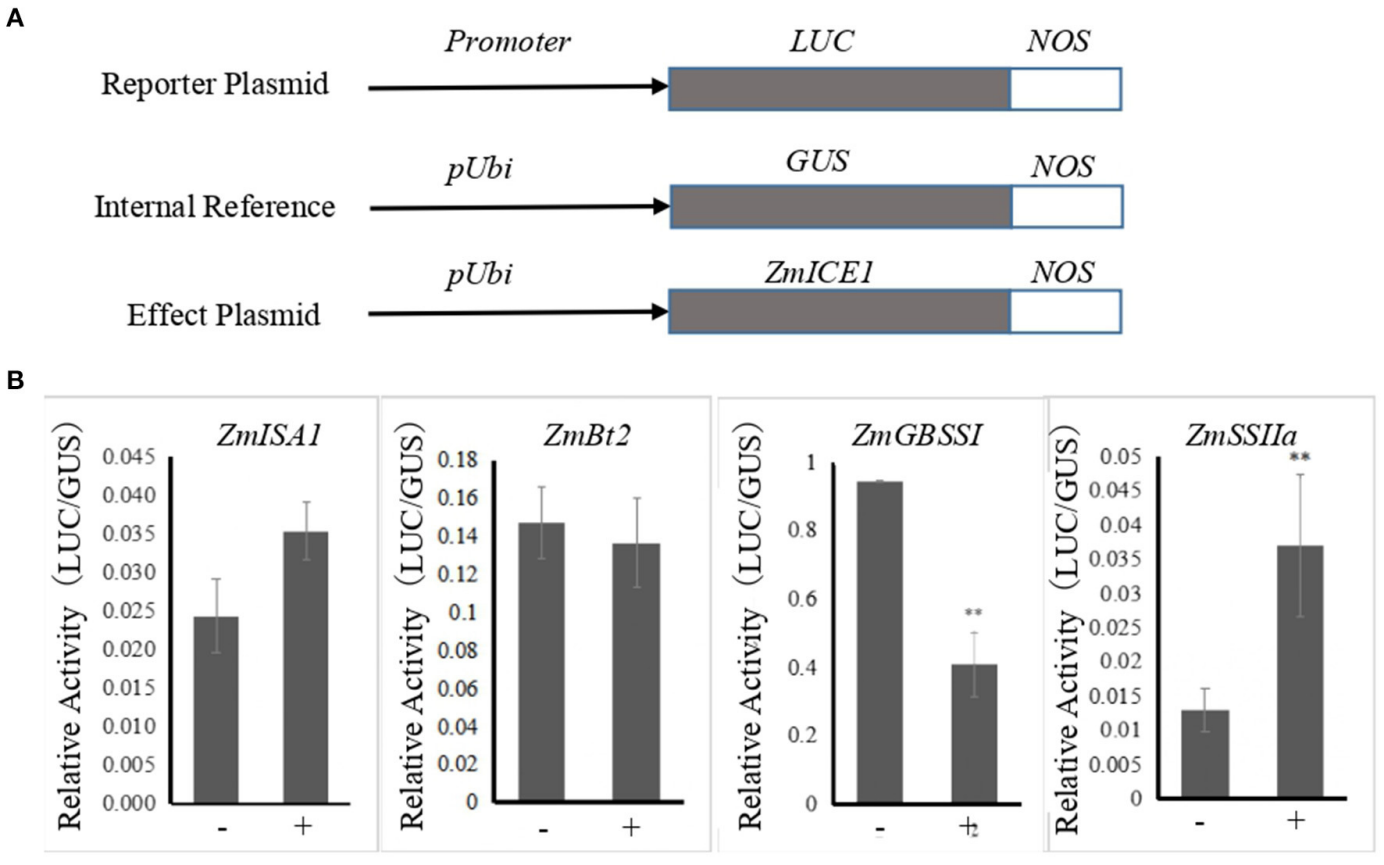
Analysis of the regulatory influence of ZmICE1 on promoter activities of genes related to starch synthesis in maize endosperm through particle bombardment-mediated transient expression assay. **(A)** Diagram of the reporter, internal reference and effect plasmids. **(B)** The promoter activities of starch synthesis genes were promoted or inhibited by ZmICE1. “−” represents the transformation containing the reporter and internal reference plasmids, and “+” transformation containing the reporter, internal reference and effect plasmids. The asterisks indicate significant differences between endosperms that are untransformed and transformed with effector plasmid (**p <0.01).

The promoters of 14 co-expressed starch synthesis-related genes contained the core binding elements recognized by the ICE subfamily, with some of the promoters having the core elements at many sites (Supplementary Table S5). This indicated that ZmICE1 may recognize and bind to them. Yeast one-hybrid assay showed that ZmICE1 binds to the promoters of 6 starch biosynthetic genes. To further study its regulatory effect on the expressions of these starch synthesis related genes, transient gene expression assays were performed, and the relative activities of β-glucuronidase (GUS) and luciferase (LUC) were measured. The reporter, internal reference and effector plasmids were co-transformed into the maize endosperm as shown in Figure 6, except for *ZmPul* and *ZmSBEI* promoters that exhibited incredibly low promoter activity. The results showed that ZmICE1 could significantly enhance the promoter activity of *ZmSSIIa*. Conversely, *ZmGBSSI* promoter activity was significantly inhibited by ZmICE1. No obvious influence was observed on the activities of *ZmBt2* and *ZmISA1* promoters. These data suggest that ZmICE1 can regulate the expressions of *ZmSSIIa* and *ZmGBSSI*, and therefore participate in starch biosynthesis.

### ZmICE1 Binds to Fragments of *ZmSSIIa* and *ZmGBSSI* Promoters

**Figure 7:**
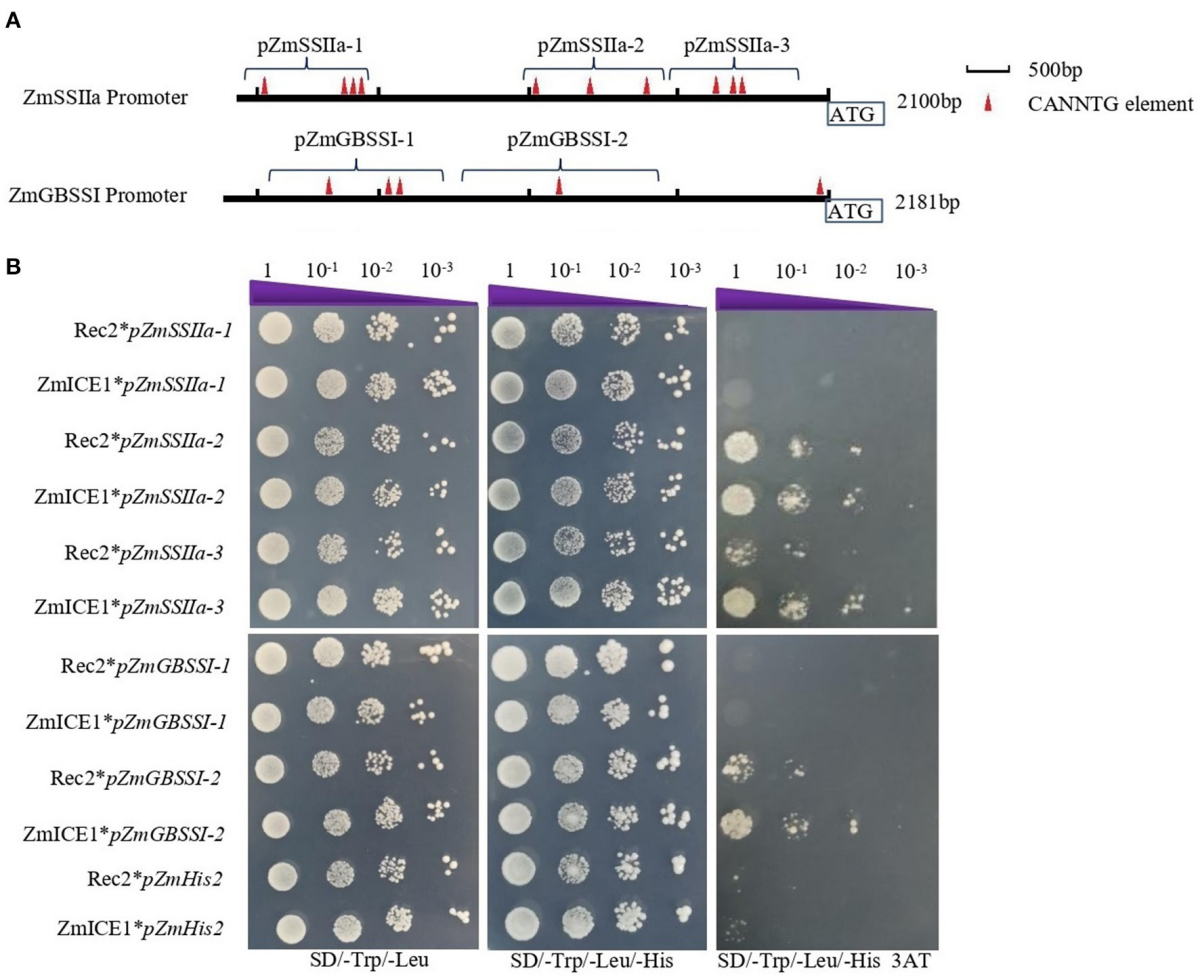
Analysis of ZmICE1 binding to *ZmSSIIa* and *ZmGBSSI* promoter fragments. **(A)** Schematic diagram of promoter fragments, showing the names, lengths, locations and the numbers of the putative core element of “CANNTG.” Red triangle represents a putative “CANNTG” core element. **(B)** Yeast one-hybrid analysis to examine binding of ZmICE1 to each promoter fragment. Transformed constructed “Rec2*pZmHis2,” “Rec2*pfragment” and “ZmICE1* *pZmHis2*” served as the negative control. “ZmICE1*pfragment” represents the experiment group.

To further explore the binding of ZmICE1 to the *ZmSSIIa* and *ZmGBSSI* promoters, we amplified 3 fragments of *ZmSSIIa* promoter (pZmSSIIa-1, a-2 and a-3), and 2 fragments of *ZmGBSSI* promoter (pZmGBSSI-1 and I-2), which contained 1–4 putative core elements of “CANNTG” in varying numbers (Figure 7A, Supplementary Table S6). The pHIS2 constructs harboring these promoter fragments were constructed for the yeast one hybrid assay. The results showed that ZmICE1 could directly bind to the fragments of pZmSSIIa-2, pZmSSIIa-3 and pZmGBSSI-2, but could not bind to the fragments of pZmSSIIa-1 and pZmGBSSI-1 (Figure 7B). Based on the positions of the binding promoter fragments, it could be suggested that the fragments that are nearer to the translation start site, are easier to be bound by ZmICE1.

## Discussion

Starch, an energy reserve in crop seeds, serves as a fuel for seedling establishment and plant regeneration. In addition, the biosynthesis and accumulations of starch in endosperm essentially affect the final grain yield and quality of the crops. Multiple functional enzymes and regulators cooperatively coordinate the processes of starch biosynthesis (Myers et al., 2000; James et al., 2003; Tetlow et al., 2004; Hennen-Bierwagen et al., 2009). Starch biosynthetic pathway, however, involves complex regulatory mechanisms that are yet to be completely simplified. Several researchers have characterized the regulations of the starch biosynthetic pathway through protein-protein interactions (Hennen-Bierwagen et al., 2008; Tetlow et al., 2008), protein modification (Tetlow et al., 2004, 2008), allosteric (Tetlow et al., 2004; Boehlein et al., 2010), and transcriptional regulations (Fu and Xue, 2010; Qi et al., 2017). Transcriptional regulation plays key roles in the molecular regulatory networks of endosperm starch biosynthesis.

It is common that the expression pattern of starch biosynthesis genes affected by transcriptional regulation to show correlations with co-expression of transcription factors and their target genes. Therefore, co-expression analysis serves as an effective research method that is widely employed in preliminary functional analysis and identification of regulators (Persson et al., 2005; Hirai et al., 2007). For example, Arabidopsis Myb28 and Myb29 are two transcription factors identified by co-expression analysis, which can regulate glucosinolate biosynthesis (Hirai et al., 2007). RSR1, which negatively regulates rice starch synthesis, was also found using co-expression analysis (Fu and Xue, 2010). In maize, co-expression and expression pattern analyses had also been used to identify transcriptional regulators of starch biosynthesis. Our previous work on ZmNAC36, which is co-expressed with starch biosynthesis genes, was revealed as a key transcription factor that regulates the transcription of genes related to starch synthesis in maize (Zhang et al., 2014). Meanwhile, many other factors directly involved in the transcriptional regulation of starch synthesis were not identified by co-expression analysis, but their expression patterns are also related to those of starch synthetic genes. For example, *OsbZIP58* also participated in the regulation of rice endosperm starch biosynthesis and showed similar expression profiles to those of starch biosynthetic genes (Wang et al., 2013). *ZmbZIP91, ZmDof3, ZmMYB14*, and *ZmNAC126* have also been identified as highly expressed transcription factors in maize endosperm which was consistent with the expression pattern of genes encoding specific enzymes related to starch synthesis (Chen et al., 2016; Qi et al., 2017; Xiao et al., 2017, 2021). Meanwhile, there may be interactions between transcription factors, which further increase the complexity of transcriptional regulation in starch biosynthesis (Kawakatsu et al., 2009; Zhang et al., 2016).

ZmICE1 was highly expressed in maize endosperm during grain filling and therefore, may function as a transcriptional regulator of starch biosynthesis. Previous reports showed that AtICE1 significantly induced the expression of cold-related genes in *Arabidopsis*, and played a crucial role in tolerance to freezing stress (Chinnusamy et al., 2003; Lu et al., 2017). It is also an important transcription factor that controls male fertility by affecting anther dehydration (Wei et al., 2018), and determines the depth of primary seed dormancy in *Arabidopsis* (MacGregor et al., 2019). In maize, previous studies reported that ZmICE1 participated in the development of maize kernel and endosperm, and nutrition metabolism through interaction with ZmO11/ ZmZHOUPI (Grimault et al., 2015). Meanwhile, ZmO11 serves as the central hub of the regulatory network for endosperm development and nutrient metabolism in maize by regulating different functional genes and transcription factors (Feng et al., 2018), suggesting that ZmICE1 may play a significant role in the regulation of starch biosynthesis in maize endosperm. Combined with our results, ZmICE1 is a typical bHLH transcription factor, and showed co-expression with genes related to starch synthesis and *ZmO11*. ZmICE1 can bind to *ZmGBSSI* and *ZmSSIIa* promoters and regulate their activity, indicating its involvement in the transcriptional regulation of genes encoding starch synthases. Generally, ZmGBSSI is mainly responsible for the extension of amylose, and ZmSSIIa is involved in the elongation of amylopectin chains (Nelson and Pan, 1995). Considering the functional roles of ZmICE1 in the regulations of *ZmGBSSI* and *ZmSSIIa*, we speculate that ZmICE1 is involved in the transcriptional regulations of maize starch biosynthesis, and may main affects grain quality by altering starch composition and structure. The regulatory functions of ZmICE1 for starch quality need to be investigated in further study. So ZmICE1 may be an important transcription factor with multiple functions, and involved in the transcriptional regulation of stress (Chinnusamy et al., 2003), kernel development (Verma et al., 2020) and starch biosynthesis in maize endosperm. The regulatory mechanism of ZmICE1 may also be complicated through interaction with other regulatory factors to participate in the various functions. However, this study only investigated the involvement of ZmICE1 in the regulation of endosperm starch synthesis.

## Conclusions

ZmICE1 is a transcription factor that contains the bHLH domain and is well conserved in plants, which has pleiotropic effects on stress, seed development and dormancy. In the study, ZmICE1 was identified through maize genome annotation database, expression profiling and co-expression analysis. We speculated that ZmICE1 was relevant to the transcriptional regulation of starch biosynthesis in maize endosperm and might affect the final yield and quality. Our study showed that ZmICE1 had transcriptional activation activity and was localized in the nucleus. Moreover, ZmICE1 could significantly regulate the expressions of *ZmSSIIa* and *ZmGBSSI* by directly binding to their promoters. Those make it possible to regulate the yield and quality of crops by affecting the starch quantity and structure. In the present preliminary study, ZmICE1 was identified as a candidate regulator that could participate in the transcriptional regulation of maize starch synthesis. More studies are required to exemplify the regulatory mechanism and the role of ZmICE1 in the modulating seed and endosperm development in maize.

## Data Availability Statement

The raw data supporting the conclusions of this article will be made available by the authors, without undue reservation.

## Author Contributions

HL, YW, and YH conceived the project and designed the experiments. YW, LL, and YL performed the experiments. HL, XW, and BW conducted bioinformatics analysis. YH contributed plant materials. HL, QX, and BA wrote and revised the manuscript. All authors read and approved the final manuscript.

## Funding

This work was funded by the Natural Science Foundation of China (No. 31601317) and the National Key Research and Development Program of China (2021YFF1000304).

## Conflict of Interest

The authors declare that the research was conducted in the absence of any commercial or financial relationships that could be construed as a potential conflict of interest.

## Publisher's Note

All claims expressed in this article are solely those of the authors and do not necessarily represent those of their affiliated organizations, or those of the publisher, the editors and the reviewers. Any product that may be evaluated in this article, or claim that may be made by its manufacturer, is not guaranteed or endorsed by the publisher.
