# Supplementary material for: Pleiotropic ZmICE1 Is an Important Transcriptional Regulator of Maize Endosperm Starch Biosynthesis
Source: Front Plant Sci. 2022 Jul 22;13:895763. doi: 10.3389/fpls.2022.895763 (PMC9355408; doi:10.3389/fpls.2022.895763)
Supplement: Supplementary file 2 [file Data_Sheet_1.docx]

Table S2: The PCC values for maize starch biosynthetic related genes with *ZmbHLH167* (*O11*) and *ZmbHLH175*(*ZmICE1*)

| Maize starch biosynthetic related genes | Transcription Factors | |
| --- | --- | --- |
|  | ZmbHLH167(O11) | ZmbHLH175(ZmICE1) |
| ZmSSIIb-2 | -0.325349037 | -0.3071063 |
| ZmSSIIIb-1 | -0.265016378 | -0.267239472 |
| ZmAGPS1 | -0.219939891 | -0.22373298 |
| ZmGBSSIIa | -0.222419605 | -0.221020605 |
| ZmISA3 | -0.251145553 | -0.216860975 |
| ZmPHOH | -0.161598403 | -0.143669603 |
| ZmAGPLS2 | -0.140951167 | -0.110814741 |
| ZmSSIIc | -0.090607851 | -0.092816593 |
| ZnISA2 | 0.000214041 | 0.015896185 |
| ZmSBEIII | 0.061863559 | 0.079911361 |
| ZmAGPLS4 | 0.070204198 | 0.091417845 |
| ZmSSIV | 0.393538797 | 0.413546798 |
| ZmAGPS2 | 0.534261454 | 0.590710819 |
| ZmAGPLS3 | 0.60620747 | 0.65027463 |
| ZmSSV | 0.641567893 | 0.678102904 |
| ZmPUL | 0.67774721 | 0.735957242 |
| ZmGBSSI | 0.803123684 | 0.877524813 |
| ZmBT1 | 0.846574574 | 0.877667533 |
| ZmBt2 | 0.84788671 | 0.906429395 |
| ZmSSI | 0.874790241 | 0.912994803 |
| ZmSBEI | 0.913019355 | 0.953831706 |
| ZmPHOL | 0.935899571 | 0.954125809 |
| ZmSSIIIa | 0.956123515 | 0.954613182 |
| ZmSh2 | 0.910267304 | 0.957034883 |
| ZmISA1 | 0.94782809 | 0.967889778 |
| ZmSBEIIb | 0.948497768 | 0.970681137 |
| ZmSSIIa | 0.964042495 | 0.973309027 |

Table S3: The information of plant ICE subfamily members used for sequence characteristics analysis

| Gene Name | Gene ID | Gene Location | Transcript ID | Number of amino acids/aa | Molecular weight（MW）/Da | Isoelectric point |
| --- | --- | --- | --- | --- | --- | --- |
| AtICE1 | AT3G26744 | Chr3:9832667~9835279 | AT3G26744.1 | 494 | 53538.93 | 5.31 |
| AtICE2 | AT1G12860 | Chr1:4384304~4386651 | AT1G12860.1 | 450 | 49795.69 | 5.85 |
| OsICE1 | Os01g0928000 | Chr1:40714216~40717112 | Os01t0928000-01 | 381 | 39776.83 | 5.76 |
| OsICE2 | Os11g0523700 | Chr11:18922046~18925254 | Os11t0523700-01 | 524 | 53529.06 | 5.56 |
| SbICE1 | SORBI_3003G412200 | Chr3:71949155~71952033 | EES04142 | 375 | 39307.17 | 4.73 |
| SbICE2 | SORBI_3005G131900 | Chr6:57378736~57382568 | EES08566 | 520 | 53723.53 | 5.07 |
| SiICE1 | SETIT_001874mg | Chr5:45479675~45482445 | KQL08441 | 376 | 39175.05 | 5.12 |
| SiICE2 | SETIT_026212mg | Chr8:26248486~26252142 | KQK94747 | 526 | 54487.45 | 5.06 |
| ZmICE1 | Zm00001d042263 | Chr3:157976446~157978862 | Zm00001d042263_P001 | 418 | 43605.94 | 4.84 |
| ZmICE2 | Zm00001d049294 | Chr4:24896748~24900567 | Zm00001d049294_P001 | 518 | 53267.92 | 5.05 |
| ZmICE3 | Zm00001d007382 | Chr2:230338653~230341206 | Zm00001d007382_P001 | 519 | 53720.73 | 5.38 |

Table S4: List of collinear genes at the *ZmICE2*/*ZmICE3* loci.

| Chromosome 2 | Transcript location | Forward or reverse strand | Gene Annotation | Chromosome 4 | Transcript location | Forward or reverse strand | Gene Annotation | Ks |
| --- | --- | --- | --- | --- | --- | --- | --- | --- |
| Zm00001d007382 | Chr2:230338653~230341206 | + | Helix-loop-helix DNA-binding domain | Zm00001d049294 | Chr4:24896748~24900567 | + | Helix-loop-helix DNA-binding domain | 0.26 |
| Zm00001d007381 | Chr2:230304901~230306029 | + | - | Zm00001d049288 | Chr4:24524855~24525593 | + | - | 0.40 |
| Zm00001d007379 | Chr2:230226142~230227194 | + | Belongs to the Casparian strip membrane proteins (CASP) family | Zm00001d049285 | Chr4:24400546~24401198 | + | Belongs to the Casparian strip membrane proteins (CASP) family | 0.36 |
| Zm00001d007378 | Chr2:230222235~230223698 | + | Belongs to the UDP-glycosyltransferase family | Zm00001d049282 | Chr4:24379653~24381203 | + | Belongs to the UDP-glycosyltransferase family | 0.31 |
| Zm00001d007372 | Chr2:230050799~230054726 | + | Belongs to the peptidase S10 family | Zm00001d049276 | Chr4:24150377~24153918 | + | Belongs to the peptidase S10 family | 0.17 |
| Zm00001d007366 | Chr2:229771474~229779655 | + | Haloacid dehalogenase-like hydrolase | Zm00001d049153 | Chr4:17983937~17992461 | + | Haloacid dehalogenase-like hydrolase | 2.46 |
| Zm00001d007360 | Chr2:229671848~229673506 | + | Armadillo beta-catenin repeat family protein | Zm00001d049258 | Chr4:23374699~23376345 | + | Armadillo beta-catenin repeat family protein | 0.23 |
| Zm00001d007347 | Chr2:229287652~229288562 | + | - | Zm00001d049242 | Chr4:22485112~22485966 | + | - | 0.54 |
| Zm00001d007345 | Chr2:229255461~229261482 | + | Belongs to the short-chain dehydrogenases reductases (SDR) family | Zm00001d049161 | Chr4:18471513~18478643 | + | Belongs to the short-chain dehydrogenases reductases (SDR) family | 0.16 |
| Zm00001d007331 | Chr2:228883647~228884624 | + | - | Zm00001d049162 | Chr4:18524687~18525670 | + | - | 0.24 |
| Zm00001d007329 | Chr2:228732533~228737268 | + | DNA binding domain | Zm00001d049173 | Chr4:19025176~19028095 | + | DNA binding domain | 0.40 |
| Zm00001d007318 | Chr2:228269951~228282169 | + | Bifunctional enzyme. Involved in de novo dTMP biosynthesis. Key enzyme in folate metabolism | Zm00001d049188 | Chr4:19758469~19764796 | + | Bifunctional enzyme. Involved in de novo dTMP biosynthesis. Key enzyme in folate metabolism | 0.20 |
| Zm00001d007316 | Chr2:228082170~228082703 | + | OST3 / OST6 family, transporter family | Zm00001d049189 | Chr4:19765914~19766447 | + | OST3 / OST6 family, transporter family | 0.15 |
| Zm00001d007307 | Chr2:227403959~227408879 | + | Acts as a Mg(2 ) transporter. Can also transport other divalent cations such as Fe(2 ), Sr(2 ), Ba(2 ), Mn(2 ) and Co(2 ) but to a much less extent than Mg(2 ) | Zm00001d049092 | Chr4:15796722~15806888 | + | Acts as a Mg(2 ) transporter. Can also transport other divalent cations such as Fe(2 ), Sr(2 ), Ba(2 ), Mn(2 ) and Co(2 ) but to a much less extent than Mg(2 ) | 0.12 |
| Zm00001d007305 | Chr2:227371967~227372398 | + | - | Zm00001d049093 | Chr4:15843740~15844195 | + | - | 0.29 |
| Zm00001d007302 | Chr2:227313582~227320505 | + | Protein of unknown function (DUF616) | Zm00001d049095 | Chr4:15932046~15937565 | + | Protein of unknown function (DUF616) | 0.25 |
| Zm00001d007301 | Chr2:227279908~227283959 | + | disulfide-isomerase | Zm00001d049099 | Chr4:15973762~15978365 | + | disulfide-isomerase | 0.40 |
| Zm00001d007300 | Chr2:227248269~227260128 | + | GAT domain | Zm00001d049100 | Chr4:16064591~16085676 | + | GAT domain | 0.28 |
| Zm00001d007295 | Chr2:226993299~226999966 | + | LIM-domain binding protein | Zm00001d049125 | Chr4:16816622~16824693 | + | LIM-domain binding protein | 0.31 |
| Zm00001d007294 | Chr2:226984490~226991125 | + | Leucine Rich repeat | Zm00001d049126 | Chr4:16825855~16829607 | + | protein At4g08330, chloroplastic-like | 0.58 |

Note: Syntenic block in maize genome was analyzed by Tbtools. *ZmICE2* and *ZmICE3* genes are underlined.

Table S5: The distribution of core element “CANNTG” in the promoters of genes related to starch biosynthesis.

| Gene Name | Gene ID | Trascript ID | Total number of the core element “CANNTG” | The positions of the core element “CANNTG” in promoters |
| --- | --- | --- | --- | --- |
| ZmSSIIa | Zm00001d037234 | Zm00001d037234_T005 | 10 | -1993/-1667/-1611/-1600/-999/-760/-615/-317/-391/-281 |
| ZmSBEIIb | Zm00001d011301 | Zm00001d011301_T001 | 6 | -1904/-1832/-1663/-1523/-787/-439 |
| ZmISA1 | Zm00001d049753 | Zm00001d049753_T001 | 3 | -1082/-1000/-948 |
| ZmSh2 | Zm00001d044129 | Zm00001d044129_T001 | 11 | -1952/-1692/-1612/-1511/-1429/-1375/-1270/-1583/-896/-614/-285 |
| ZmSSIIIa | Zm00001d000002 | Zm00001d000002_T002 | 15 | -1942/-1685/-1266/-1103/-1041/-949/-871/-780/-679/-636/-388/-324/-291/-135/-76 |
| ZmPHOL | Zm00001d034074 | Zm00001d034074_T003 | 12 | -1965/-1952/-1734/-1628/-1614/-1585/-1172/-1160/-528/-485/-209/-158 |
| ZmSBEI | Zm00001d014844 | Zm00001d014844_T005 | 14 | -1932/-1678/-1421/-1373/-1059/-904/-609/-560/-472/-369/-378/-206/-253/-41 |
| ZmSSI | Zm00001d045261 | Zm00001d045261_T006 | 4 | -1067/-940/-506/-269 |
| ZmBt2 | Zm00001d050032 | Zm00001d050032_T003 | 11 | -1941/-1188/-1116/-970/-993/-714/-651/-405/-285/-250/-219 |
| ZmBT1 | Zm00001d015746 | Zm00001d015746_T001 | 17 | 2-1980/-1591/-1353/-1154/-1034/-902/-838-853/-816/-753/-601/-490/-423/-413/-367/-332/-281 |
| ZmGBSSI | Zm00001d033937 | Zm00001d033937_T002 | 5 | -1749/-1452/-1415/-898/-50 |
| ZmPUL | Zm00001d004438 | Zm00001d004438_T005 | 10 | -1844/-1611/-1591/-1547/-1537/-1333/-1168/-882/-770/-614 |
| ZmSSV | Zm00001d051976 | Zm00001d051976_T002 | 15 | -1784/-1517/-1504/-1386/-1294/-994/-909/-888/-824/-601/-538/-493/-301/-221/-79 |
| ZmAGPLS3 | Zm00001d019266 | Zm00001d019266_T002 | 11 | -1992/-1984/-1939-1447/-1403/-1312/-1194/-848/-816/-656/-566 |
| ZmAGPS2 | Zm00001d032385 | Zm00001d032385_T001 | 4 | -1795/-1710/-1030/-805 |
| ZmSSIV | Zm00001d010821 | Zm00001d010821_T002 | 6 | -1595/-1212/-1009/-822/-813/-131 |
| ZmAGPLS4 | Zm00001d033910 | Zm00001d033910_T002 | 5 | -1811/-1743/-1512/-1497/-1422 |
| ZmSBEIII | Zm00001d003817 | Zm00001d003817_T002 | 9 | -1736/-1463/-1228/-1157/-827/-801/-614/-458/-161 |
| ZnISA2 | Zm00001d038121 | Zm00001d038121_T001 | 7 | -1955/-1143/-1025/-873/-531/-363/-169 |
| ZmSSIIc | Zm00001d014150 | Zm00001d014150_T005 | 12 | -1940/-1910/-1757/-1744/-1642/-1603/-1488/-1364/-1064/-1019/-804/-526 |
| ZmAGPLS2 | Zm00001d039131 | Zm00001d039131_T007 | 10 | -2000/-1836/-1719/-1242/-1192/-658/-581/-398/-373/-16 |
| ZmPHOH | Zm00001d042842 | Zm00001d042842_T003 | 7 | -1585/-690/-656/-649/-357/-338/-163 |
| ZmISA3 | Zm00001d020799 | Zm00001d020799_T003 | 5 | -1810/-1650/-1558/-674/-506 |
| ZmGBSSIIa | Zm00001d019479 | Zm00001d019479_T004 | 10 | -1370/-958/-933/-846/-799/-647/-402/-392/-28/-21 |
| ZmAGPS1 | Zm00001d005546 | Zm00001d005546_T002 | 5 | -1749/-1044/-858/-313/-248 |
| ZmSSIIIb-1 | Zm00001d026337 | Zm00001d026337_T007 | 17 | -1770/-1743/-1687/-1550/-1480/-1111/-1101/-1080/-1010/-962/-887/-866/-803/-146/-271/-138/-38 |
| ZmSSIIb-2 | Zm00001d018033 | Zm00001d018033_T001 | 4 | -1909/-1826/-1734/-987 |

Note: The promoter fragment of 2000bp upstream of the translation start site was obtained and used for the core element analysis. The location of the first base, upstream of the translation start site, is recorded as -1.

Table S6: Information of *ZmSSIIa* and *ZmGBSSI* promoter fragments

| Name | The length of promoter fragment/bp | Location of promoter fragment | Number of core element “CANNTG” in the promoter fragment | Positions of core elements “CANNTG” | Sequence of sense primer | \| Sequence of antisense primer \| \| --- \| |
| --- | --- | --- | --- | --- | --- | --- | --- |
| pZmSSⅡa-1 | 562 | -2090_-1528 | 4 | -1993_-1987;-1667_-1661; -1611_-1605; -1600_-1594 | GACTCACTATAGGGCGAATTCCGACAAATGGTTGCCTCG | CGGATCGATTCGCGAACGCGTGAAGAAGGGCATCACGCTAG |
| pZmSSⅡa-2 | 504 | -1032_-528 | 3 | -999_-993;-760_-754;-615_-609 | GACTCACTATAGGGCGAATTCGCAATCTGTTGAAACCGAG | CGGATCGATTCGCGAACGCGTGAGAGCAATAGAGTGGCGTG |
| pZmSSⅡa-3 | 383 | -509_-126 | 3 | -391_-385;-317_-311;-281_-275 | GACTCACTATAGGGCGAATTCCTTATACAAGATCCATACC | CGGATCGATTCGCGAACGCGTGAGTGAGGAGGGTTTAC |
| pZmGBSS1-1 | 520 | -1902_-1382 | 3 | -1750_-1744;-1452_-1446;-1415_-1409 | GACTCACTATAGGGCGAATTCCGACGGATAACGGAAACC | CGGATCGATTCGCGAACGCGTACCCAGGAAGCACAGGAGA |
| pZmGBSS1-2 | 577 | -1220_-645 | 1 | -898_-892 | GACTCACTATAGGGCGAATTCTTTCATAGGGCTCAGGATTTAG | CGGATCGATTCGCGAACGCGTTCGGTTCTCGGCTGCTTG |

Note: The restriction enzyme sites of EcoRI and MluI in primers are shown in red. Homologous recombination sequences of primers are shown in blue.
