# Supplementary figures and images for: Pleiotropic ZmICE1 Is an Important Transcriptional Regulator of Maize Endosperm Starch Biosynthesis
Source: Front Plant Sci. 2022 Jul 22;13:895763. doi: 10.3389/fpls.2022.895763 (PMC9355408; doi:10.3389/fpls.2022.895763)

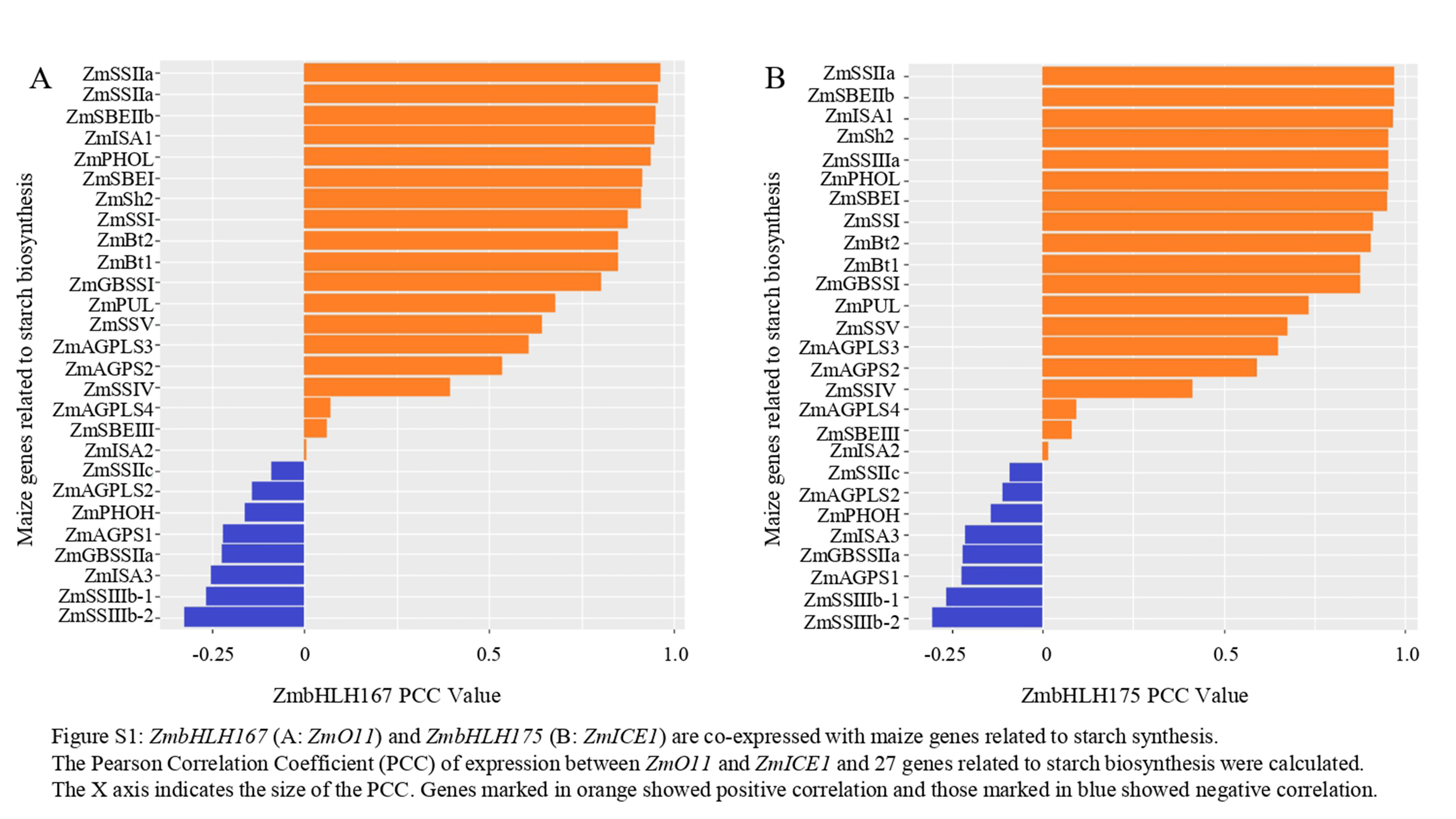

Supplement: Supplementary file 3 [file Image_1.JPG]

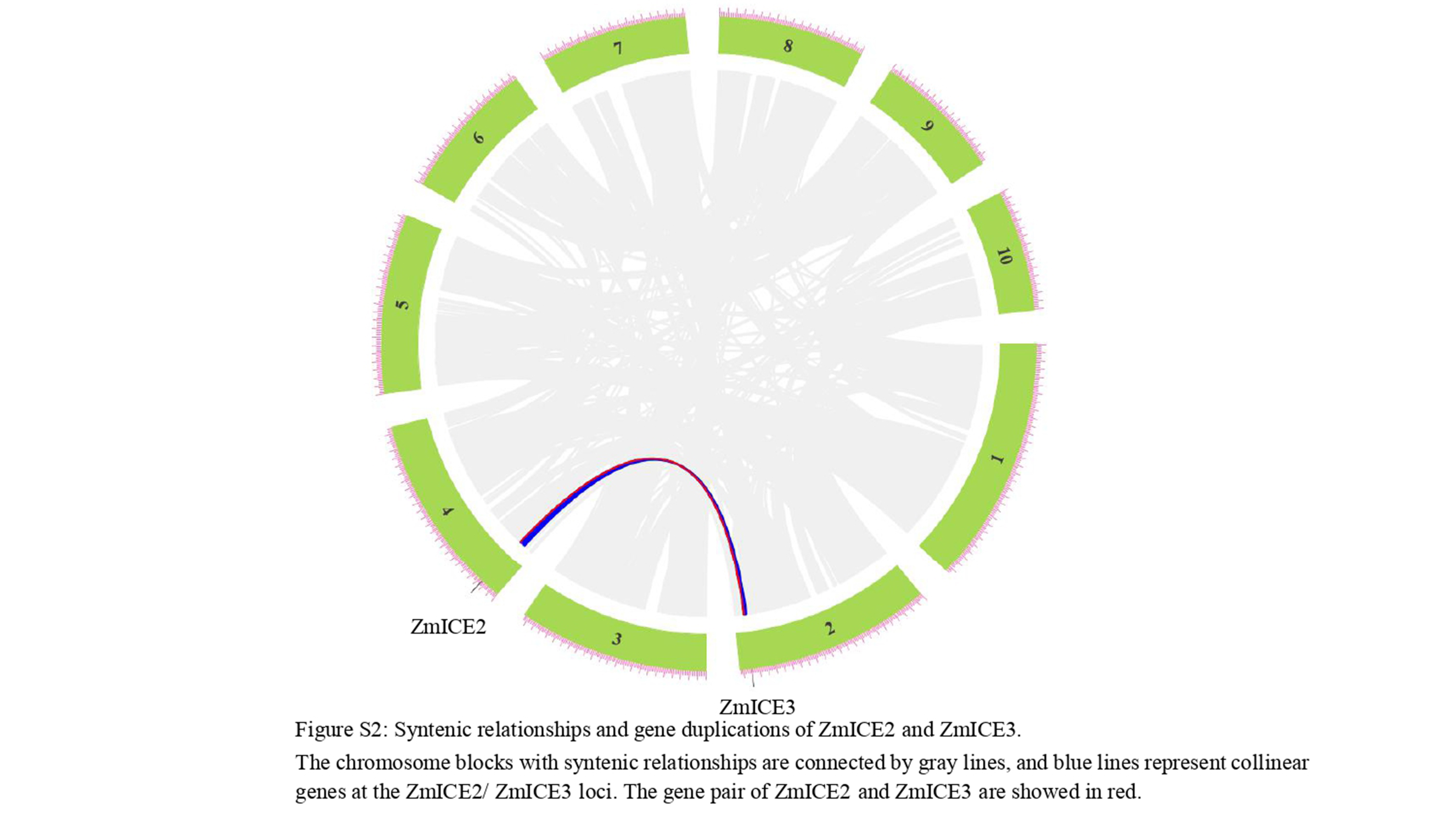

Supplement: Supplementary file 4 [file Image_2.JPG]
